# Studies on Poly(propylene fumarate-co-caprolactone diol) Thermoset Composites towards the Development of Biodegradable Bone Fixation Devices

**DOI:** 10.1155/2009/486710

**Published:** 2009-02-25

**Authors:** M. Jayabalan

**Affiliations:** Polymer Division, Biomedical Technology Wing, Sree Chitra Tirunal Institute for Medical Sciences and Technology, Thiruvananthapuram 695 012, India

## Abstract

The effect of reinforcement in the cross-linked poly(propylene fumarate-co-caprolactone diol) thermoset composites based on Kevlar fibres and hydroxyapatite was studied. Cross-linked poly(propylene fumarate-co-caprolactone diol) was also studied without any reinforcement for comparison. The reinforcing fibre acts as a barrier for the curing reaction leading to longer setting time and lesser cross-link density. The fibre and HA reinforced composites have almost the same compressive strength. Nonreinforced material undergoes greater degree of swelling. Among the reinforced materials, the hydroxyapatite reinforced composite has a much higher swelling percentage than the fibre reinforced one. The studies on in vitro degradation of the cured materials reveal hydrolytic degradation in Ringer's solution and PBS medium during aging. All the three materials are found to swell initially in Ringer's solution and PBS medium during aging and then undergo gradual degradation. Compression properties of these cross-linked composites increase with aging; HA reinforced composite has the highest compressive strength and compressive modulus, whereas the aged fibre-reinforced composite has the least compressive strength and modulus.

## 1. Introduction

Metal plates and screws have been commonly used in the operative treatment of bone fracture to stabilize the fracture site [1]. The metals, most commonly used for internal plate fixation, are 316 stainless steel, cobalt-chromium alloys, titanium and its alloys. They allow maintenance of good contact between bone fragments. Metal plates prevent primary callus formation due to the absence of mechanical stimulation at the fracture site and decrease bone density. Similarly adequate screw fixation in osteoporotic bone is a difficult and challenging surgical problem due to screw loosening and subsequent implant failure [2]. Since metal plates are more rigid than bone they transmit the majority of the stress received by the bone-plate system, resulting in localised bone atrophy and osteoporosis during latter stages of fracture healing. Stress shielding interferes with blood circulation and the weaker bone may fracture again after implant removal [3]. The eventual surgical removal of metal plates and screws after they have served their stabilisation function is, therefore, required. Thus plates which exert less stress protection on bone and more flexible fixation of fracture are of increasing interest.

An ideal fracture fixation device should have optimal mechanical properties during the two stages of healing [4]. Initially, the implant must have sufficient stiffness to allow bony union without angulations [5]. Some degree of mechanical stimulation at the fracture site is desired to allow the formation of cortical callus, the most rapid way to achieve bony union and restore the strength of the broken bone to its original level [6]. During the second stage of fracture healing, the implant must deform sufficiently under a given load to increase the strain within the bone, permitting remodelling to occur and avoid the effects of stress protection.

Bone fixation devices present a challenging area for the application of composite technology due to the nature of the process of bone formation and resorption. Bone plates of reinforced polymer composites have shown some success in the prevention of this stress protection phenomenon [7]. Fibre reinforced polymer composites of high volume fraction and perfect alignment possess a wide range of properties such as specific strength and stiffness. Biodegradable polymer matrix not only sparks new bone growth over time, but also provides mechanical strength and breaks down into natural metabolites that are then excreted from the body. The advantages that draw on the heterogeneity and anisotropy of synthetic composite biomaterials can be easily anticipated. The elastic properties of composites can be accurately controlled and be tailor-made to closely match to the elastic property of host bone tissue. Anisotropy can be used to advantage in biomedical structures which require specific stress and stiffness like longitudinal and transverse rigidities for bone replacement application. Heterogeneity can be utilised in a partially biodegradable composite implant, where the matrix will be gradually resorbed to make room for the regenerated natural tissue. The latter will grow on to the reinforcement which serves as a permanent scaffold.

Though biodegradable plates offer a viable method to overcome the adverse effects of metal plates, they are presently unsuitable for bone fixation because of insufficient mechanical properties and optimum biostability. Biodegradable plate suffers from warping, hollowing, or substantial erosion inherent with the process of degradation. To solve the problem of insufficient mechanical strength, polymers with high crystallinity have been explored [8]. Reinforcing elements such as fibres of crystalline polymers, fibres of carbon in polymeric resins, and particulate fillers have also been studied [9]. Recently interpenetrating network in a biodegradable implant was reported for improving the mechanical strength [10].

Composites of containing Kevlar polyaramide fibre or inorganic hydroxyapatite in conjunction with unsaturated polyester resins offer composites with high strength to weight ratio; such composites are more promising materials for orthopedic applications such as bone plates and screws for fracture fixation. The inorganic material Kevlar fibre is biostable and does not elicit adverse host reactions. Studies on tissue compatibility of Kevlar aramid 49 (du Pont) fibres and aramid-polymethylmethacrylate (PMMA) composites reveal tissue compatibility [11]. Salmonella-microsome assay (Ames) and mammalian cell cultures showed no evidence for any cyto- or genotoxic effects of aramid fibres have been observed with aramid fibres [12]. The inorganic hydroxyapatite is a resorbable. As a part of the development of biodegradable unsaturated polyester resins for various orthopedic uses, oligomers of poly(propylene fumarate)-based resins, which can cross-link in situ with n-vinyl pyrrolidone comonomer, were synthesised by Jayabalan et al. [13]. The paper deals with the studies on the effect of reinforcement in the cross-linked poly(propylene fumarate-co-caprolactone diol) thermoset composites on the biodegradation and biomechanical stability under simulated physiological conditions.

## 2. Materials and Methods

### 2.1. Preparation and Characterization of Oligomeric Resins

Carboxy terminated-poly(propylene fumarate-co-capro-lactone diol), PPF-PL, was synthesized by the direct esterification and cocondensation of maleic anhydride, 1-2, propylene glycol and polycaprolactone diol_2000_ at high temperature and vacuum conditions. The initial condensation reaction between maleic anhydride and 1-2, propylene glycol was followed by condensation with polycaprolactone diol_2000_ in a single continuous step. The reaction was catalyzed by sodium acetate and morpholine. The reaction product was dissolved in acetone and then washed with 25% aqueous methanol to remove unreacted reactants. The polymer was reprecipitated in petroleum ether, filtered, and dried under vacuum to highly viscous, yellowish brown, and transparent resin, and analysed by IR and 1HNMR spectral studies.

### 2.2. Preparation and Characterization of Cross-Linked Materials

Comonomer n-vinyl pyrrolidone (Fluka Chemie AG, Buchs, Switzerland) was used for curing of the oligomers. Kevlar fibre 49 (Dupond De Nemours and Company, Singapore) and hydroxyapatite of (125-*μ*m particle size) were used as reinforcing fillers for polymeric composites. The setting time and exothermic temperature of the oligomeric resin and n-vinyl pyrrolidone (n-vp) with and without reinforcing fillers were determined as per ISO 5833/1-1999 E Standard. The average temperature was determined by taking half the sum of ambient temperature and exothermic setting temperature. The time taken to reach the average temperature was found as setting time.

Cross-linked and reinforced composite material, PPF-PL/nVp/KF, was prepared by cross-linking PPF-PL oligo-meric resin (6 g) with n-vinyl pyrrolidone (3.768 g) and reinforcing filler crystalline Kevlar fibre (2% the weight of resin) in the presence of initiator benzoyl peroxide (0.03 g) and accelerator N,N-dimethyl aniline (1 *μ*l) as per the formulation given in Table 1. Cross-linked and reinforced composite material with 125-*μ*m hydroxyapatite (equal to the weight of resin), PPF-PL/nVp/HA, and cross-linked PPF-PL (without reinforcing fillers), PPF-PL/nVp, was also prepared for comparative study.

Surface properties of the cross-linked materials were analyzed using attenuated total reflection (ATR) spectroscopy. The IR spectrum of the cross-linked materials was recorded using a Nicolet Impact 410 FT-IR instrument with ATR spectroscopic accessory. The surface morphology was investigated using scanning electron microscope (SEM). The samples were fractured by immersing in liquid nitrogen and the cross-sectional area of the fractured sample was analysed after critical point drying (CPD) and gold sputtering. 

The effective cross-link density (mol/cm^3^) and the number of the average molecular weight between cross-links (*M*_*c*_) of the cured material were determined as reported elsewhere [14]. Initially the swelling coefficient (*θ*) was determined by subjecting the polymer sheet to attain equilibrium swelling in different solvents with different solubility parameters. The solvent in which the polymer showed maximum swelling was used for determining the swelling coefficient (*Q*). In the present polymers, maximum swelling was observed in dimethyl formamide. Therefore, the accurately weighed polymer materials were allowed to swell in it for 2 days and the increase in weight was measured. The swelling coefficient (*Q*) is the ratio of volume of solvent in the swollen polymer to that of swelled polymer,

(1) $$\begin{array}{ll} & \text{Swelling coefficient}(Q)\\ & \;\;=\frac{\text{Weight}\;\text{ of}\;\text{ the}\;\text{ solvent}\;\text{ in}\;\text{ swollen}\;\text{ polymer }}{\text{Weight}\;\text{ of}\;\text{ the}\;\text{ swelled}\;\text{ polymer}}\\ & \;\;\;\times \frac{\text{Density}\;\text{ of}\;\text{ polymer}}{\text{Density}\;\text{ of}\;\text{ solvent}}. \end{array}$$

The volume fraction of the polymer in the swollen polymer (*V*_*r*_) was calculated from swelling coefficient. The solubility parameter of dimethyl formamide solvent (*δ*_s_), which imparts maximum swelling, was taken as the solubility parameter of the polymer (*δ*_p_). The effective cross-link density and molecular weight between cross-links [$\overset{¯}{Mc}=1/\gamma$] of swollen polymers were determined by using modified Flory-Rehner's equation,

(2) $$\text{Crosslink Density},\gamma =\frac{-\left[V_{r}+\chi V_{r^{2}}+\ln\left(1-V_{r}\right)\right]}{d_{r}\;\;V_{o}\left(V_{r^{1/3}}-V_{r}/2\right)},$$

where *V*_*r*_ = 1/(1 + *θ*) is the volume fraction of the polymer, *d*_*r*_ is density of the polymer, *χ* is polymer–solvent interaction parameter (lattice constant 0.34 for *δ*_s_ = *δ*_p_ (s-solvent, p-polymer)), and *V*_*o*_ is molar volume of the solvent.

The cross-linked materials were also molded in the form of cylindrical pellets of 6 mm in diameter by 12 mm height for mechanical testing. Compressive testing was done with an Instron model series IX automated materials testing system −7.43.00 at cross-head speed of 5 mm/min at room temperature in accordance with the ASTM F451-86 specification. Hardness was measured based on the resistance of the cured materials to the penetration of an intender of a specific dimension measured in the calibrated dial of a Shore D durometer as per ASTM standard D 2240-81. The data reported were the mean of 12 measurements.

The thermal behaviour of the cross-linked materials was studied by thermogravimetric analysis (TGA) and differential thermal analysis (DTA). The samples were heated to a maximum temperature range of 500°C at the heating rate of 10°C/min in inert nitrogen atmosphere. A Universal VI. 12E TA instrument was used.

### 2.3. Studies on Biodegradation of Cross-Linked Materials

Dimensional stability of the material was evaluated by measuring the percentage of swelling. Cross-linked materials were weighed accurately (*W*_1_) and then placed in distilled water at room temperature. The swollen cross-linked materials at equilibrium swelling were removed to measure the final weight (*W*_2_). The percentage of swelling was determined by

(3) $$\text{Percentage of swelling}=[\frac{W_{2}-W_{1}}{W_{1}}]\times 100.$$

Percentage of swelling gives a measure of dimensional stability of the material.

The biodegradation of the cross-linked materials was studied by in vitro aging in various simulated biological media. The media used were phosphate buffered saline (PBS) of pH 7.4 and Ringer's solution of pH 8.3, prepared as per standard procedure [15]. 0.2 M disodium hydrogen phosphate (28.4 g of anhydrous Na_2_HPO_4_. in 1000 mL) and 0.2 M sodium dihydrogen phosphate (27.6 g of NaH_2_PO_4_·H_2_O in 1000 mL) in 0.9% saline solution were prepared separately and mixed in the ratio 77:23 (v/v) to prepare PBS (pH 7.4). Ringer's solution was prepared by dissolving sodium chloride (9 g), sodium hydrogen carbonate (0.42 g), calcium chloride (0.24 g), and glucose (1 g) in 1000 mL distilled water. Pellets (dimension 6 mm × 12 mm (*n* = 6)) were accurately weighed and immersed in different media (25 mL) in screw capped vials and kept at 37°C. In alternate days, the samples were taken out and weighed for 7 days without changing the media. The percentage of weight change was plotted against duration of exposure. The compressive testing was also carried at wet condition as in the previous case and variation in compressive strength and modulus was evaluated. The surface morphology of the aged samples in simulated biological media was investigated using scanning electron microscope (SEM).

## 3. Results and Discussion

A major advantage of the use of biodegradable implants is that the removal of such implants from the body after they have fulfilled their purposes is not necessary. This has been accomplished by the incorporation of hydrolytically labile or enzymatically cleavable bonds leading to nontoxic degradation products [16]. The degradation of the majority of the polyesters is thought to occur by bulk hydrolysis of the ester bonds [17]. Bulk degradation as observed in most polyesters is characterized by the penetration of water into the polymer matrix, where after hydrolysis of the ester bonds starts and the molecular weight to decrease. Weight loss will start when the molecular weight of the polymer chains has sufficiently decreased [18]. For the hydrolytic degradation of aliphatic polyesters, the presence of comonomers plays an important role on the properties of the cured polyesters. Unsaturated poly(propylene fumarate) copolymerised with biodegradable macrodiols, namely, poly(caprolactone diol) displays variable properties as biodegradable materials.

### 3.1. Synthesis and Characterisation of Poly(Propylene Fumarate-co-Caprolactone Diol) Resin (PPF-PL)

**Scheme 1:**
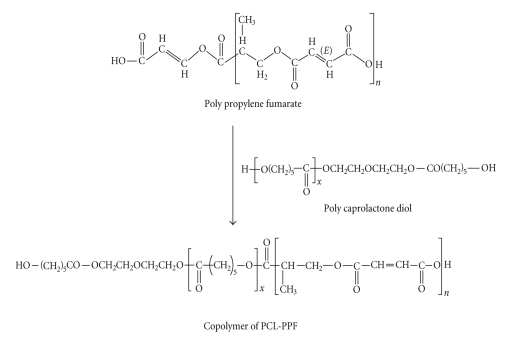
Synthesis of poly(propylene fumarate-co-caprolactone diol) resin.

Oligomeric resin, poly(propylene fumarate-co-caprolactone diol), was synthesized. The equilibrium condensation reactions between maleic anhydride and 1,2 propylene diol along with polycaprolactone diol (2000) in a typical experimental procedure resulted in the formation of a random oligomer as shown in Scheme 1.

**Figure 1:**
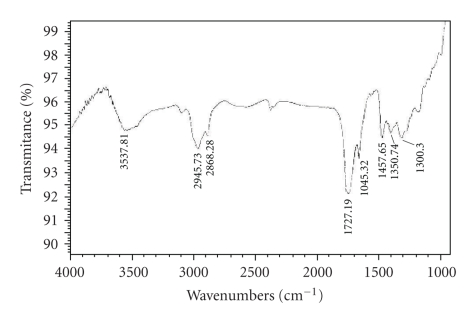
FT-IR spectrum of poly(propylene fumarate-co-caprolactone diol).

**Figure 2:**
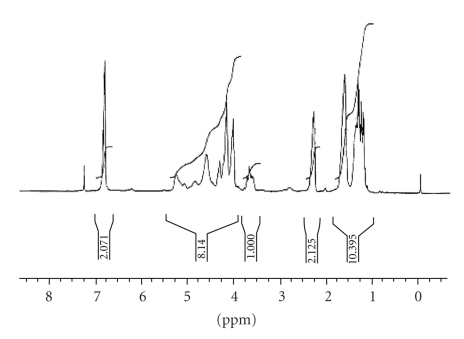
'H-NMR spectrum of poly(propylene fumarate-co-caprolactone diol).

The formation of oligomeric copolymer, PPF-PL, was confirmed by FT-IR spectrum. The spectral analysis indicates the presence of ester group (C=O stretching) at 1727.2 cm^−1^ and unsaturated double bond ( − CH=CH − ) at 1645.3 cm^−1^ and 982.03 cm^−1^ in oligomeric copolymer (see Figure 1). The spectral analysis also indicates frequencies at 3537.8 cm^−1^ (O–H, hydrogen bonded), 2945.7 cm^−1^, and 2868.3 cm^−1^ (aliphatic C–H), 1457.7 cm^−1^ (–CH_2_–), 1300.3 cm^−1^ (O − H), 1155.1 cm^−1^ ( − C − O − ) for the presence of polycaprolactone unit. The ^1^H NMR spectrum of oligomeric resin PPF-PL is given in Figure 2. The peaks were assigned based on the similar oligomer containing poly(propylene fumarate) and polyethylene glycol [19]. The olefinic protons absorb at 6.8 ppm (doublet) and the methyl protons absorb at 1.2–1.4 ppm (multiplet). The protons of the terminal propyl methine group and the methylene group absorb at 4.1 ppm and 4.0 ppm, respectively, whereas that of the internal propyl methylene group and methine group absorb at 4.1–4.3 ppm (multiplet) and 5.2–5.3 ppm, respectively. The ethylene group of caprolactone unit gives a peak at 3.65 ppm, whereas the ethylene group near ester gives a peak at 3.7 ppm. Peaks near 4.0–4.2 ppm are due to the hydrogen of − O − CH_2_ − which is coming from the polycaprolactone blocks in the polymer chain [20]. The UV-visible spectrum of the resin in THF showed absorption maximum around 235 nm corresponding to *n* → Π* and Π → Π* transitions due to C=O and C=C groups.

The pale yellow viscous resin has shown an acid value of 102.81, a higher value compared to that of similar copolymeric resins containing polyethylene glycols with molecular weight 600, 1000, and 2000 instead of polycaprolactone diol 2000 [13]. The studies on inherent viscosity of resin revealed the inherent viscosity as 0.572 (dl/g).

### 3.2. Setting Characteristics of the Oligomeric Resin in the Presence of Reinforcing Fillers

**Scheme 2:**
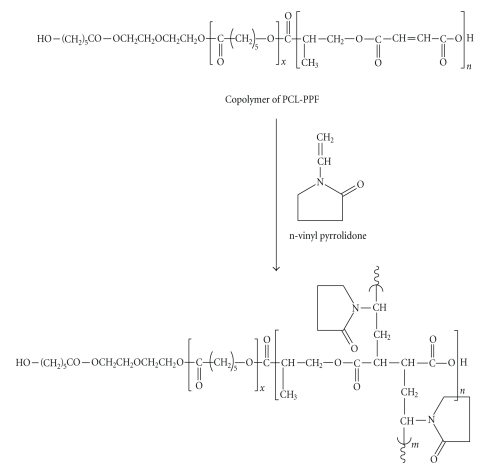
Crosslinking reaction of poly(propylene fumarate-co-caprolactone diol) with n-vinyl pyrrolidone.

Setting characteristics of the oligomeric resin with comonomer n-vinyl pyrrolidone, with and without reinforcing fillers (Kevlar and hydroxyapatite), was carried out as per the formulation given in Table 1. The setting reaction involved is a cross-linking reaction of unsaturated poly(propylene fumarate-co-caprolactone diol) with n-vinyl pyrrolidone as given in Scheme 2. Cross-linking of PPF-PL with n-vinyl pyrrolidone takes place via free radical polymerisation mechanism. After the addition of the vinyl monomer n-vinyl pyrrolidone, cross-linking begins on the introduction of an initiator, benzoyl peroxide, and accelerator, N,N-dimethyl aniline. The cross-linking takes place between the double bonds of the vinyl groups of n-vinyl pyrrolidone to form random copolymer. The data on setting characteristics of resin with and without fillers is given in Table 2. The setting times for PPF-PL/nVp and PPF-PL/nvp/HA were found to be the same while the setting time of PPF-PL/nvp/KF has increased greatly even with the presence of just 2% Kevlar fibre. This indicates that fibres can act as a barrier for the reaction between PPF-PL and n-vinyl pyrrolidone while hydroxyapatite (1.250 *μ*) would not. The setting temperature and maximum exothermic temperatures were found to be more or less the same irrespective of the presence and nature of the reinforcing fillers.

### 3.3. Evaluation of Cross-Linked Materials

#### 3.3.1. Cross-Link Density and Molecular Weight between Cross-Links

All the present cross-linked materials were found to swell in common organic solvent such as toluene, hexane, ethanol, tetrahydrofuran, and dimethyl acetamide. Maximum swelling was observed in DMA, as the solubility product of DMA was close to that of the polymer. The molecular weight between cross-links and the cross-link density is given in Table 3. A higher cross-link density is observed in the case of nonreinforced cross-linked material in comparison with the reinforced cured composites. From this, it is clear that the reinforcing fillers act as a barrier for the cross-linking reaction between the unsaturated polyester and the vinyl monomer, n-vp. The cross-link density in the case of Kevlar fibre reinforced composites was found to be less than that of hydroxyapatite reinforced composites. This is attributed to the more heterogeneity of fibre reinforced composites compared to that of the particulate filler reinforced composites. The relation between swelling and cross-link density is directly proportional up to a limiting cross-link density above which it is indirectly proportional. Since the Kevlar fibre inhibits the cross-linking reaction between PPF-PL and n-vinyl pyrrolidone, cross-link density is rated in the following order: cured PPF-PL/nvp > PPF-PL/nvp/HA > PPF-PL/nvp/KF.

#### 3.3.2. IR Spectral and SEM Studies

**Figure 3:**
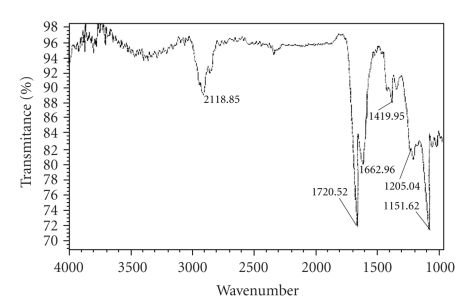
FT-IR spectrum of cross-linked PPF-PL/nvp material.

**Figure 4:**
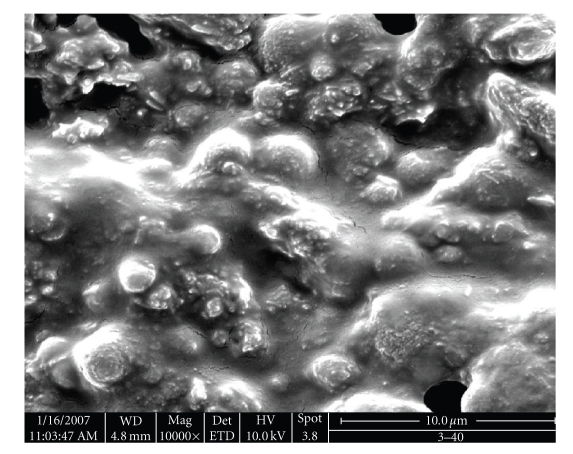
SEM pictures of cross-linked PPF-PL/nvp/HA material.

The IR spectrum of PPF-PL/nvp cross-linked material (see Figure 3) showed the disappearance of the peak at 1645 cm^−1^ due to cross-linking at the − CH=CH − group. The peak at 1420 cm^−1^ can be attributed to the aliphatic –C ≡ N– vibrations and those at 1662 cm^−1^ and 1720 cm^−1^ can be attributed to the vibrations of − C=O − group of nvp and fumarate, respectively. The SEM analysis of cross-linked material reveals the presence of microvoids in virgin composites and the lack of strong interfacial adhesion in Kevlar fibre reinforced composites in comparison with hydroxyapatite filler reinforced composite. Hydroxyapatite filler reinforced composite exhibits good interfacial adhesion. Representative SEM microphotograph of hydroxyapatite filler reinforced composite is given in Figure 4.

#### 3.3.3. Mechanical Properties

The compressive test data for the reinforced polymeric composites with fibre and hydroxyapatite are given in Table 4. Cross-linked PPF-PL/nvp material has shown a compressive strength of 177.25 MPa and compressive modulus 1157.96 MPa. Reinforced polymeric composites with Kevlar fibre and hydroxyapatite have shown almost the same compressive strength, 174.14 MPa and 173.67 MPa, respectively. However, the compressive modulus of PPF-PL/nvp/HA increased drastically compared to PPF-PL/nvp/KF and PPF-PL/nvp. The high modulus of PPF-PL/nvp/HA system may be due to the high filler content (resin: HA = 1:1). The compressive modulus of PPF-PL/nvp system is greater than that of fibre reinforced composites. This may be due to low cross-link density of the latter as well as low interfacial bond strength at the resin-fibre interface. However, the Shore D hardness is found to be greater in the case of PPF-PL/nvp/KF (55) when compared to that of PPF-PL/nvp/HA (40.75) and PPF-Pl/nvp (45.13).

#### 3.3.4. Thermal Analysis

**Figure 5:**
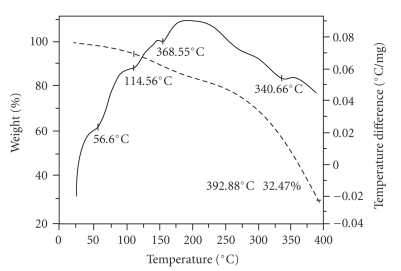
TGA-DTA thermograms of cross-linked PPF-PL/nvp material.

**Figure 6:**
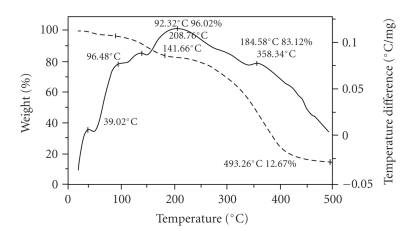
TGA-DTA thermograms of cross-linked PPF-PL/nvp/KF material.

**Figure 7:**
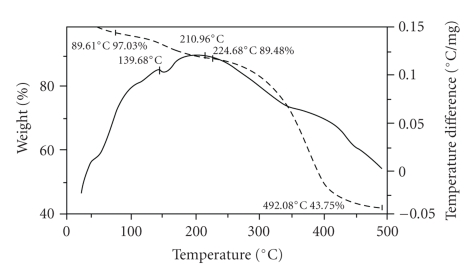
TGA-DTA thermograms of cross-linked PPF-PL/nvp/HA material.

The TGA-DTA curves of PPF-PL/nvp, PPF-Pl/nvp/KF, and PPF-Pl/nvp/HA are given in Figures 5, 6, and 7. The weight percentage of the remaining resin, after the decomposition at 400°C, in PPF-PL/nvp, PPF-PL/nvp/KF, and PPF-PL/nvp/HA is 30, 24, and 53, respectively. Thus, the hydroxyapatite reinforced composite has greater thermal stability than the nonreinforced polymer, which has greater thermal stability than the Kevlar fibre reinforced composite. The softening temperature (*T*_*m*_) of PPF-PL/nvp is found to be higher (56.80°C) than the reinforced composites (see Table 5). Obviously, this is due to the higher cross-link density of the nonreinforced cross-linked material when compared to that of the reinforced composites. The softening temperature of PPF-PL/nvp/KF and PPF-PL/nvp/HA is found to be the same. All the three cross-linked materials show predominant decomposition peaks, suggesting multistage degradation.

#### 3.3.5. Swelling Behaviour

The swelling percentage value calculated in terms of the weight of water absorbed per 100 g of dry polymer is a measure of dimensional stability [10]. As the swelling index value increases, dimensional change increases. Among the cross-linked materials, PPF-PL/nvp system has shown higher percentage of swelling, 6.25 compared to reinforced composites, 1.775 for PPF-PL/nvp/KF and 4.8319 for PPF-PL/nvp/HA. PPF-PL/nvp/KF has the least percentage of swelling indexes due to low cross-link density and low interfacial bond strength at fibre-resin interface. PPF-PL/nvp/HA has higher dimensional stability in comparison with that of PPF-PL/nvp. This is due to its higher cross-link density in comparison with other cross-linked materials.

### 3.4. In Vitro Biodegradation

**Figure 8:**
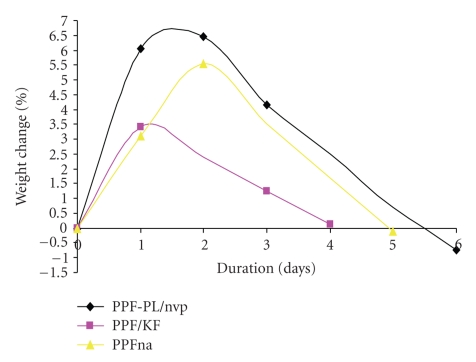
Degradation of cross-linked materials in Ringer's solution.

**Figure 9:**
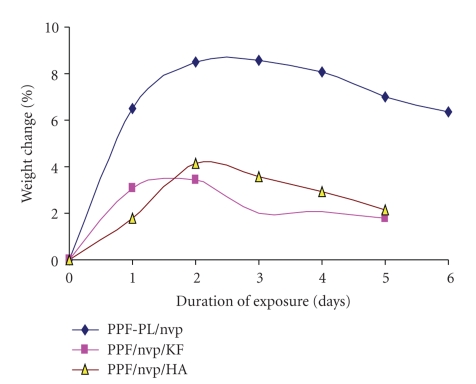
Degradation of cross-linked materials in PBS medium.

**Figure 10:**
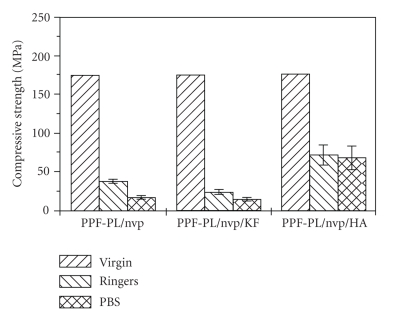
Change in compressive strength of aged crosslinked materials.

**Figure 11:**
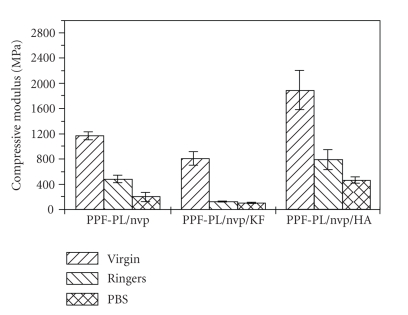
Change in compressive modules of aged crosslinked materials.

**Figure 12:**
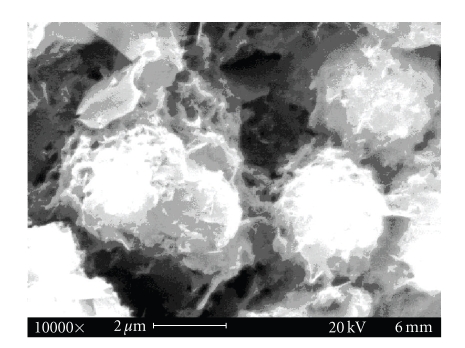
Degradation and formation of scaffold structure in cross-linked PPF-PL/nvp/HA material in PBS medium.

The in vitro biodegradation of the cross-linked materials was studied using Ringer's solution and PBS medium at 37°C. The weight loss and pH data clearly indicate a hydrolytic degradation in all the cross-linked materials. The pH of the solution decreased and stabilised to more or less a constant value (see Table 6). The alkaline hydrolysis of the ester linkages in the cross-linked polymer PPF-PL/nvp leads to the release of acidic components, which could further cause acid-catalysed ester group hydrolysis. All the three cross-linked materials are found to swell initially in both media (Ringer's and PBS) and then undergo gradual degradation (see Figures 8 and 9). The degradation is found to be much faster in Ringer's solution than in PBS medium. Maximum swelling is found in the case of nonreinforced cured PPF-PL/nvp when compared to the reinforced cured composites. This might be because of the higher cross-link density of PPF-PL/nvp than that of the reinforced cross-linked materials. Similarly, PPF-PL/nvp/HA which has a higher cross-link density when compared to that of PPF-PL/nvp/KF is found to swell more. The compressive test data of these aged cross-linked materials (7 days aging) is shown in Tables 7 and 8. Among the three aged cross-linked materials, hydroxyapatite reinforced composite has the highest compressive strength and compressive modulus, whereas fibre reinforced composite has the least compressive strength and modulus in both media (see Figures 10 and 11). All the three cross-linked materials are found to have greater compressive strength and modulus in Ringer's solution than in PBS medium. The present studies reveal the slow degradation of the polymeric matrix; such degradation leads to the formation of a scaffold structure that has been revealed by the SEM analysis. Representative SEM microphotograph for the aged cross-linked-PPF-PL/nvp/HA material in PBS medium is given in Figure 12. Kevlar fibre remains unaffected in the medium during aging. The scaffold formation in the cross-linked is more favourable for bone growth and anchorage with the host bone.

## 4. Conclusion

The data on setting studies and cross-link density reveal that the Kevlar fibre acts as a barrier for the completion of cross-linking reaction. The compressive modulus of the hydroxyapatite containing composite is higher than that of the nonreinforced one, which is higher than the fibre reinforced composite. Hardness follows the reverse order. Thermal studies of cured reinforced composites exhibit a mild softening at around 50°C, whereas the nonreinforced composites soften at around 56°C. From the swelling data of the cured materials, it is observed that the nonreinforced material has the highest swelling, which is due to its high cross-link density. Among the reinforced materials, the hydroxyapatite reinforced composite has a much higher swelling percentage than the fibre reinforced one. The studies on in vitro degradation of the cured materials reveal hydrolytic degradation in Ringer's solution and PBS medium during aging. All the three materials are found to swell initially in both the aging media and then undergo gradual degradation. The lowering of pH of the aging media, dimensional instability, and the weight change supports faster degradation. The compressive test data indicate that the aged hydroxylapatite reinforced composite has the highest compressive strength and compressive modulus, where as the aged fibre reinforced composite has the least compressive strength and modulus.

In conclusion, these polymers are suitable biodegradable materials for application as bioabsorbable polymeric fixation devices. The properties of the composites can be altered by varying the amount of the filler content, depending on the intended end use. Kevlar fibre reinforced composites show a much lower rate of weight change when compared to the hydroxyapatite reinforced composites. The rate of biodegradation is the highest in the case of the nonreinforced polymer. So depending on the required lifespan of the biomaterial, the appropriate filler can be chosen.

## Figures and Tables

**Table 1:** Formulation of the cross-linked material.

Cross-linked material	Weight of the constituent of the cross-linked material (g)					
	PPF-PL resin	n-Vinyl pyrrol idone	Kevlar fibre	Hydroxyapatite	Benzoyl peroxide	N,N-dimethyl aniline (*μ*l)
PPF-PL/nvp	6	3.768	—	—	0.03	1
PPF-PL/nvp/KF	6	3.768	0.12	—	0.03	1
PPF-PL/nvp/HA	6	3.768	—	6	0.03	1

**Table 2:** Setting characteristics.

Cross-linked material	Setting time (minutes)	Setting temperature (°C)	Maximum exothermic temperature (°C)
PPF-PL/nvp	3.50	38.7	44.2
PPF-PL/nvp/KF	7.75	39.5	47.0
PPF-PL/nvp/HA	3.75	39.5	48.0

**Table 3:** Physical properties of cross-linked products of PPF-PL-based resin.

Cross-linked	Density	Swelling	Cross-link density (*γ*)	Molecular weight between
material	(g/cc)	coefficient (*θ*)	(mol/cc) (×10^−2^)	cross-links ($\overset{¯}{M_{c}}$)
PPF-PL/nvp	1.2232	0.1960	1.2369	80.847
PPF-PL/nvp/KF	1.2176	0.2741	0.9064	110.329
PPF-PL/nvp/HA	1.550	0.1977	0.9681	103.593

**Table 4:** Mechanical properties of cross-linked products of PPF-based resin.

Cross-linked material	Load at maximum (kN)	Compressive strength (MPa)	Compressive modulus (MPa)	Hardness shore D
PPF-PL/nvp	5.012 ± 0.0036	177.254 ± 0.1315	1157.958 ± 63.97	45.13 ± 0.83
PPF-PL/nvp/KF	4.924 ± 0.002	174.135 ± 0.066	794.683 ± 105.438	55 ± 0.93
PPF-Pl/nvp/HA	4.910 ± 0.003	173.669 ± 0.093	1850.886 ± 311.361	40.75 ± 0.89

**Table 5:** Differential thermal analysis data.

Cross-linked materials	Softening temperature (°C) *T*_*m*_	Decomposition temperature (°C)		
		Td_1_	Td_2_	Td_3_
PPF-PL/nvp	56.80	160.55	275	340.66
PPF-PL/nvp/KF	50	140	208.76	358.34
PPF-PL/nvp/HA	50	139.68	210.96	365.24

**Table 6:** In vitro biodegradation of cross-linked products in hydrolytic media.

Cross-linked materials	Ringer's solution			PBS medium		
	Initial pH	Final pH	Weight change after 7 days (%)	Initial pH	Final pH	Weight change after 7 days (%)
PPF-PL/nvp	8.3	6.21	6.4633	7.4	7.13	8.7379
PPF-PL/nvp/ KF	8.3	5.42	3.4395	7.4	7.09	3.5347
PPF-PL/nvp/ HA	8.3	5.2	6.0203	7.4	7.1	4.1783

**Table 7:** Mechanical properties of cross-linked materials aged in Ringer's solution.

Cross-linked materials	Load at maximum (kN)	Compressive strength (MPa)	Percentage loss of compressive strength	Compressive modulus (MPa)	Percentage loss of compressive modulus
PPF-PL/nvp	1.060 ± 0.07	37.491 ± 2.46	78.84	473.207 ± 57.76	78.84
PPF-PL/nvp/KF	0.654 ± 0.09	23.117 ± 3.13	86.72	97.459 ± 13.78	86.72
PPF-PL/nvp/HA	1.959 ± 0.36	69.300 ± 12.69	60.09	713.691 ± 116.36	60.09

**Table 8:** Mechanical properties of cross-linked materials aged in PBS medium.

Cross-linked materials	Load at maximum (kN)	Compressive strength (MPa)	Percent age loss of compressive strength	Compression modulus (MPa)	Percentage loss of compressive modulus
PPF-PL/nvp	0.474 ± 0 .07	16.754 ± 2.32	90.55	182.129 ± 74.16	84.27
PPF-PL/nvp/K F	0.384 ± 0 .07	13.587 ± 2.39	92.19	78.723 ± 10.13	90.09
PPF-PL/nvp/HA	1.849 ± 0 .42	65.388 ± 15.03	62.23	421.877 ± 51.07	77.21

## References

[1] Tergesen T, Apalset K (1988). The influence of different degrees of stiffness of fixation plates on experimental bone healing. *Journal of Orthopaedic Research*.

[2] Strømøse K, Kok WL, Høiseth A, Aiho A (1993). Holding power of the 4.5 mm AO/ASIF cortex screw in cortical bone in relation to bone mineral. *Injury*.

[3] Roman SR, Garcia PG (1991). Partially biodegradable polyacrylic-polyester composites for internal bone fracture fixation. *Biomaterials*.

[4] Dodge HS, Cady GW (1972). Treatment of fractures of the radius and ulna with compression plates. *The Journal of Bone and Joint Surgery. American*.

[5] Woo SLY Internal fixation plates for fracture management: new design concepts.

[6] McKibbin B (1978). The biology of fracture healing in long bones. *The Journal of Bone and Joint Surgery. British*.

[7] Tenino AJ, Folmer RCH (1987). The clinical use of plastic plates for osteosynthesis in human fractures. *Clinical Materials*.

[8] Shikinami Y, Okuno M (1999). Bioresorbable devices made of forged composites of hydroxyapatite (HA) particles and poly-L-lactide (PLLA)—part I: basic characteristics. *Biomaterials*.

[9] Thomson RC, Yaszemski MJ, Powers JM, Mikos AG (1998). Hydroxyapatite fiber reinforced poly(*α*-hydroxy ester) foams for bone regeneration. *Biomaterials*.

[10] Hasirci V, Lewandrowski KU, Bondre SP, Gresser JD, Trantolo DJ, Wise DL (2000). High strength bioresorbable bone plates: preparation, mechanical properties and in vitro analysis. *Bio-Medical Materials and Engineering*.

[11] Henderson JD, Mullarky RH, Ryan DE (1987). Tissue biocompatibility of kevlar aramid fibers and polymethylmethacrylate, composites in rabbits. *Journal of Biomedical Materials Research*.

[12] Wening JV, Langendorff U, Delling G, Marquardt H, Hoffmann M, Jungbluth KH (1989). First results on biocompatibility, cyto- and genotoxicity testing of aramid fibers in the rabbit. *Unfallchirurgie*.

[13] Jayabalan M, Thomas V, Sreelatha PK (2000). Studies on oIy(propylene fumarate-co-ethylene glycol) based bone cement. *Bio-Medical Materials and Engineering*.

[14] Thomas V, Jayabalan M A new generation of high flex life polyurethane urea for polymer heart valve—studies on in vivo biocompatibility and biodurability.

[15] Celin D, Thomas V, Jayabalan M (2000). Diethylene glycol acrylate-n-vinyl pyrrolidone copolymer resins for bone cement applications. *Indian Journal of Engineering and Materials Sciences*.

[16] Doherty PJ, Williams RL, Williams DF, Lee AJC (1992). *Bioinaterials-Tissue Interfaces*.

[17] Holland SJ, Tighe BJ, Gould PL (1986). Polymers for biodegradable medical devices. 1. The potential of polyesters as controlled macromolecular release systems. *Journal of Controlled Release*.

[18] Leenslag JW, Pennings AJ, Bos RRM, Rozema FR, Boering GR (1987). Resorbable materials of poly(L-lactide): VII. In vivo and in vitro degradation. *Biomaterials*.

[19] Suggs LJ, Payne RG, Yaszemski MJ, Alemany LB, Mikos AG (1997). Synthesis and characterization of a block copolymer consisting of poly(propylene fumarate) and poly(ethylene glycol). *Macromolecules*.

[20] Ural E, Kesenci K, Fambri L, Migliaresi C, Piskin E (2000). Poly(D,L-cactide/*ɛ*-caprolactone)/hydroxyapatite composites. *Biomaterials*.

